# Evaluation of neurogenic bladder outlet obstruction mimicking sphincter bradykinesia in male patients with Parkinson’s disease

**DOI:** 10.1186/s12883-021-02153-4

**Published:** 2021-03-19

**Authors:** Tianying Xing, Jinghong Ma, Tongwen Ou

**Affiliations:** 1 Department of Urology, Xuanwu Hospital, Capital Medical University, No.45 Changchun Street, Xicheng, Beijing, 100053 People’s Republic of China; 2 Department of Neurology, Xuanwu Hospital, Capital Medical University, Beijing, 100053 People’s Republic of China

**Keywords:** Parkinson’s disease, Delayed sphincter relaxation, Sphincter bradykinesia, Voiding dysfunction, Urodynamics

## Abstract

**Background:**

Lower urinary tract symptoms are one of the most common groups of non-movement symptoms in patients with Parkinson’s disease (PD). Storage symptoms are well-acknowledged, but neurogenic voiding dysfunction caused by PD remains a knowledge gap. This study aimed to evaluate the neurogenic bladder outlet obstruction in male patients with PD and its clinical significance.

**Methods:**

Male patients who were diagnosed with PD and underwent urodynamic studies were retrospectively reviewed. The patients with prostate size < 30 ml and bladder outlet obstruction index ≥40 were included in the study. Lower urinary tract symptoms were evaluated by International Prostate Symptom Score (IPSS). Free flowmetry was performed and post void residual (PVR) volume was measured by ultrasound at follow-up.

**Results:**

Six patients were included in the final analysis. The mean age was 68.2 and the mean movement symptom duration was 70.7 months. The patients had a mean IPSS of 12.5 and mean PVR volume of 70.8 ml. All patients had slow stream but none of them reported significant voiding difficulty. Urodynamic studies showed the delayed urinary sphincter relaxation and the special trace pattern. After a mean follow-up of 20 months, they had a mean IPSS of 12.5 and mean PVR volume of 73.3 ml. None of them complained of significant voiding difficulty at follow-up.

**Conclusion:**

The delayed urinary sphincter relaxation is a rare but repeatable phenomenon in male patients with PD. It is unlikely to cause disturbing voiding dysfunction, as reported by the patients, and does not progress prominently during the course of PD. Further studies are needed to investigate the nature of this special type of neurogenic BOO and whether it is peculiar to PD in a larger patient cohort.

**Supplementary Information:**

The online version contains supplementary material available at 10.1186/s12883-021-02153-4.

## Background

Parkinson’s disease (PD) is a progressive neurodegenerative disorder, characterized by bradykinesia, rigidity, tremor and postural instability. Besides motor symptoms, non-motor symptoms also occur frequently in PD patients, which further affect patients’ quality of life [1]. Lower urinary tract symptoms (LUTS) are one of the most prominent categories among non-motor symptoms, with the reported incidence ranging from 27 to 85% in different studies [2]. The main pathological change of PD is the dopaminergic neuron loss in substantia nigra above pons. Thus, it impairs the overall inhibitory effect on micturition reflex [3]. As a result, LUTS in PD patients primarily manifest as storage symptoms, including nocturia, frequency and urgency. Voiding symptoms like hesitancy, slow stream or feeling of incomplete emptying are generally less common than storage symptoms, and they are less severe than those in patients with multiple system atrophy (MSA), which often masquerades as PD but has an aggressive nature [4]. Currently, less researches focus on voiding dysfunction in patients with PD.

Detrusor underactivity or bladder outlet obstruction (BOO) underlie the mechanism of voiding symptoms. Although detrusor contractility often decreases in patients with PD compared to healthy controls, it is still preserved to a certain degree. Thus, PD patients mostly maintain an acceptable voiding efficiency and low post void residual (PVR) volume, making it a differential parameter between PD and MSA [5]. At the meantime, PD mostly affects the elderly, overlapping the age group with high morbidity of benign prostatic hyperplasia (BPH). Therefore, BPH often brings bias when understanding the pathophysiology of micturition in PD or evaluating BOO specifically caused by PD. Neurogenic BOO, i.e. non-prostatic BOO in PD patients still draws less attention.

Neurogenic BOO is typically caused by detrusor sphincter dyssynergia (DSD), which is frequently observed in patients with spinal cord injury and MSA, etc. The incidence of DSD in PD is actually low (0–3%) in the current opinion, as early studies probably included MSA due to the lack of diagnostic criteria and the difficulty in differentiation [6]. Given this reason, it requires further investigation to understand other forms of neurogenic BOO in PD patients. Currently, the neurogenic BOO caused specifically by PD remains as a knowledge gap. Whether the neurogenic BOO in PD causes disturbing voiding dysfunction (like in MSA) or whether it progresses with the disease course of PD (as motor symptoms do) is largely unknown. In our study, we analyzed male PD patients without BPH to minimize the prostate bias. We aimed to identify the neurogenic BOO in these patients and to understand its clinical significance.

## Materials and methods

### Study design

We retrospectively reviewed male patients who were initially diagnosed as PD by movement disorder specialists and underwent urodynamic study from January 2013 to August 2020 at our single institute. The diagnosis of PD (at baseline or follow-up) was made in accordance with the clinical diagnostic criteria of the International Parkinson and Movement Disorder Society [7]. To evaluate the neurogenic BOO specifically caused by PD, we included patients with obstructed voiding during the pressure flow study, defined as bladder outlet obstruction index (BOOI) ≥ 40. And to minimize the prostate bias, we included the patients only whose prostate size was < 30 ml without protruded median lobes evaluated by transrectal prostate ultrasound. Exclusion criteria were patients with other neurological conditions that could cause neurogenic bladder (e.g. cerebral vascular disease, lumbar spondylosis); patients with secondary cause of parkinsonism, urinary tract infection, urinary lithiasis, prostate cancer and pelvic surgery history; patients with prostate size larger than 30 ml or unknown; patients who had history of urethral injuries; patients who could not show detrusor contraction during the urodynamic study. Follow-up data was acquired from the movement disorder specialists to confirm the correct diagnosis of PD. If the patients’ diagnosis was revised, then they were not included in the analysis.

### Urodynamic study

All urodynamic procedures were in accordance with the guidelines of the International Continence Society and performed using the same protocol (Triton, Laborie, ON, Canada). Before each study, the urologists recorded the patients’ history of LUTS, the International Prostate Symptom Score (IPSS), the brief history of PD and anti-parkinsonian medications. None of the patients were taking anti-muscarinic, α-receptor blocker within 2 weeks prior to the urodynamic study. After free flowmetry, a 7-Fr dual lumen urodynamic loop catheter (COOK, IN, USA) was inserted to measure the intravesical pressure. A 10-Fr balloon catheter (COOK, IN, USA) was inserted into rectum to measure the abdominal pressure. The surface electrodes were placed at the external anal sphincter for electromyography (EMG) to evaluate sphincter function during the urodynamic study if EMG was done. A filling cystometry was performed with the room-temperature saline at a filling rate of 10–50 ml/min when a patient was in a sitting position. A pressure flow study was also performed when the patient was in a sitting position, but the patient might be asked to stand up and void repeatedly if necessary. PVR volume was measured after the pressure flow study. Bladder outlet obstruction was defined as BOOI ≥40 (BOOI = Detrusor pressure at maximum flow [PdetQmax] — 2 x maximum flow rate [Qmax]). Detrusor contractility was evaluated by bladder contractility index (BCI, BCI = PdetQmax + 5 x Qmax). A free flowmetry was done if the patient visited the urologist for follow-up. PVR volume was measured by ultrasound after free-flowmetry.

## Results

### Patient demographics

A total of 78 male patients with PD were reviewed. Twenty-nine patients had prostate size below 30 ml. Only 6 of them had BOOI ≥40 and they were included in the analysis. The most common reasons for exclusion were prostate size over 30 ml or diagnosis revision during follow-up. Patients’ demographics were summarized in Table 1. The mean age of the patients was 68.2 years and the mean duration of movement symptoms was 70.7 months. Three patients had the movement symptom duration no more than 12 months and Hoehn & Yahr grade 1. They were not on anti-parkinsonian medication at the time of urodynamic studies. The other 3 patients had the movement symptom duration more than 108 months and Hoehn & Yahr grade 3 (at on-phase), with motor complications due to the advanced stage and long-term levodopa usage. Generally, the 6 patients had mild to moderate LUTS evaluated by the IPSS (mean 12.5, ranging from 8 to 15). Patient 2, 3, 5 and 6 had higher IPSS, primarily because they had severer storage symptoms including nocturia, urgency and frequency. For voiding symptoms, all patients had slow stream. But none of them complained of hesitancy, intermittency, straining to void or feeling of incomplete emptying, and they denied voiding difficulty.

**Table 1:** Patient demographics at baseline

Patient	Age (years)	Movement symptom duration (months)	Hoehn & Yahr scale	LEDD (mg)	IPSS	Comment
1	66	6	1	0	8	Newly diagnosed
2	58	10	1	0	14	–
3	73	132	3	1330	15	On off and wearing off phenomena
4	63	12	1	0	9	–
5	74	108	3	1198	14	On off and wearing off phenomena
6	75	156	3	1830	15	On off and wearing off phenomena

*LEDD* levodopa equivalent daily dose, *IPSS* international prostate symptom score

### Urodynamic study

Filling cystometry showed detrusor overactivity (DO) existed in 4 of the 6 patients, and 3 of them had significantly reduced bladder capacity after a long disease duration (Table 2). On pressure flow study, the 6 patients had mean BOOI of 61.7 ± 8.9 and mean BCI of 100.5 ± 12.2. The mean PVR volume was 70.8 ml. Only patient 1 had a large PVR volume of 265 ml, while all the other 5 patients had PVR volume less than 50 ml.

**Table 2:** Urodynamic data of the patients

Patient	DO	Capacity (ml)	PdetQmax (cmH_2_O)	Qmax (ml/s)	BOOI	BCI	Detrusor contractility^a^	PVR (ml)
1	–	510	60	4	52	80	Weak	265
2	+	290	69	5	59	94	Weak	30
3	+	170	85	6	71	115	Normal	25
4	–	330	66	7	52	101	Normal	45
5	+	200	76	6	64	106	Normal	40
6	+	100	82	5	72	107	Normal	20

*DO* detrusor overactivity, *PdetQmax* detrusor pressure at maximum urine flow, *Qmax* maximum urine flow, *BOOI* bladder outlet obstruction index, *BCI* bladder contractility index, *PVR* post void residual

^a^ Detrusor contractility was evaluated by Schäfer’s nomogram

**Fig. 1:**
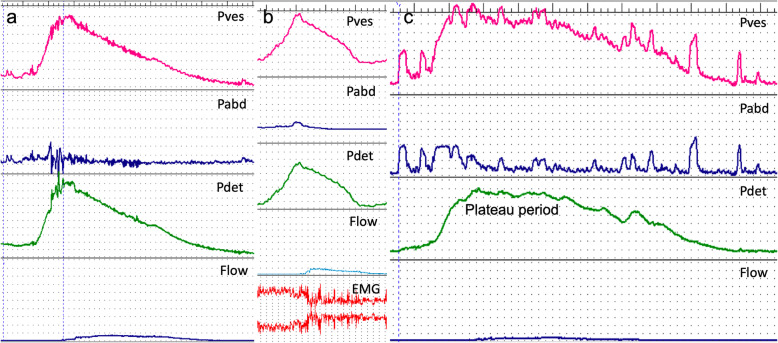
Urodynamic traces a. The pressure flow trace of a male patient with Parkinson’s disease showed the continuous drop of detrusor pressure after urine flow began and a low flow rate; b. The pressure flow trace of a male patient with Parkinson’s disease showed similar features. EMG showed the delayed sphincter relaxation; c. The pressure flow trace of an age-matched male patient with benign prostatic obstruction showed detrusor pressure exhibited a “plateau period”. Pves: vesical pressure; Pabd: abdominal pressure; Pdet: detrusor pressure; EMG: electromyography

Of note, the pressure flow study traces of these 6 patients shared the unique abnormal characteristics: the detrusor could initiate contraction normally and reach high opening pressure but then dropped continuously with a consistent slow flow rate (Fig. 1a, b). Due to the abnormal special trace pattern, pressure flow studies were repeated but displayed the same shape. This was hardly seen in the pressure flow traces of patients with BPH, in which the detrusor typically reached high opening pressure and sustained with a “plateau period” to maintain proper pressure and efficient voiding (Fig. 1c). Neither was this special trace pattern common in other male patients with PD. EMG was recorded in 4 of the 6 patients, which showed that the sphincter did not relax properly at the initiation of detrusor contraction until the detrusor contracted to a higher pressure, mimicking sphincter bradykinesia (Fig. 1b). A previous study reported that sphincter bradykinesia might be peculiar to PD. We reviewed our urodynamic database and did not discover this special urinary sphincter dysfunction in patients with MSA.

### Follow-up data

Table 3 showed the follow-up data. The mean follow-up time was 20 months. All patients were on anti-parkinsonian medication and none of them received surgery for PD or LUTS. No diagnosis revision was made during the follow-up period. The mean IPSS was 12.7 and the mean PVR volume was 73.3 ml. The IPSS and PVR volume were comparable to the baseline. Only patient 1 was receiving α-receptor blocker regularly. Although he had improved Qmax, he still had a large PVR volume of 245 ml. Meanwhile, he still did not complain voiding difficulty or a sense of incomplete voiding. Other 5 patients used anti-muscarinic agents intermittently to relieve their storage symptoms. They did not report worsened voiding function either.

**Table 3:** Follow up data of the patients

Patient	Follow-up time (months)	IPSS at follow-up	Qmax at follow-up	PVR at follow-up
1	18	7	10	245
2	24	12	7	40
3	25	14	6	35
4	20	12	8	40
5	30	16	5	55
6	3	15	5	25

*IPSS* international prostate symptom score, *Qmax* maximum urine flow, *PVR* post void residual

## Discussion

Patients with PD frequently suffer from LUTS, including both voiding and storage symptoms. Voiding disorders draw less attention in this patient group, since their voiding problems are much less severe than those of in patients with MSA [4]. Although detrusor underactivity is not rare in patients with PD, neurological BOO caused specifically by PD has not been well documented and little is known about its clinical significance. We reviewed the male PD patients with minimal prostate bias and found that neurogenic BOO existed in patients with PD probably due to the delayed relaxation of sphincter, mimicking sphincter bradykinesia. This was a rare but repeatable abnormality in male patients with PD. Also, follow-up data showed that sphincter bradykinesia did not cause progressing voiding dysfunction during the course of PD.

Detrusor overactivity with proper sphincter relaxation is the most common urodynamic finding in patients with PD, but sphincter bradykinesia was rare [8]. A study by Pavlakis et al. reported 2 PD patients with sphincter bradykinesia (using CO_2_ as the filling medium and perineal needle EMG) [8]. Another study showed 5 male patients with had non-prostatic BOO, using the criteria of prostate size ≤30 ml [9]. Our study, using the same criteria, showed 6 male PD patients with neurogenic BOO. Of the 6 patients, only patient 1 accepted cystoscopy examination because of his large PVR volume, but there were no positive findings on bladder or bladder neck. Other patients refused to have further cystoscopy as they did not regard voiding dysfunction as their main problem. This special BOO was then largely because of the delayed sphincter relaxation, as none of the patients met the exclusion criteria for some common reasons of BOO. Also, the sphincter dysfunction was similar but was not detrusor sphincter dyssynergia, as the sphincter did not contract when the detrusor was contracting, nor did the EMG show augmented or persisting sound. Furthermore, some patients might voluntarily contract pelvic floor muscles simultaneously with detrusor contraction because of DO (to control urine leakage) or pain after catherization, causing pseudo-dyssynergia. However, those patients could relax their sphincters properly after permitting to void (See Additional file 1). Our patients did not meet this condition as they voided without contracting their pelvic floor muscles after permission was given. Our study reinforced the previous studies on the rarity of this voiding abnormality. However, due to the limited sample size of all studies, we may infer that the neurogenic BOO exists in a larger proportion of male patients with PD, as it is less likely to cause severe voiding dysfunction; it is also easily confounded with BPH or ignored by patients.

Previous studies have identified the similar sphincter dysfunction in patients with PD, but little is known about its clinical significance. All of the 6 patients in our study did not report obstructed voiding as their main concern at the time of urodynamic studies, although their pressure flow studies were obviously aberrant. The only voiding symptom reported by the patients was slow stream, but none of them complained of straining to void, hesitancy, intermittency or feeling of incomplete emptying. This could account for voiding difficulty not being a main problem in these 6 patients. The patient with the short follow-up time (3 months) was the most recent patient who had PD history for 13 years and movement complications as well because of the levodopa usage. Thus, little is questioned about his diagnostic accuracy of PD. Also, he had kept the preserved voiding function since the onset of PD. It is less likely that his voiding function would deteriorate progressively. For the other 5 patients, they did not report significant worsened voiding function after a mean follow-up time of 23.4 months, evaluated by IPSS and free flowmetry. Their diagnosis also remained PD at follow-up. Therefore, we speculate that the non-prostatic BOO caused by sphincter bradykinesia in these PD patients might not have an aggressive nature as it brought limited disturbance on voiding and did not deteriorate significantly during the course of PD.

Compared with the effect of dopaminergic agents on storage symptoms, less is known about their effects on urinary sphincter. Uchiyama et al. reported that levodopa facilitated voiding 1 h after medication by enhancing detrusor contractility in patients with advanced PD. But to a less extent, levodopa also increased urethral resistance [10]. In rats, urethral resistance decreased by activating D2 dopamine receptor [11]. Hence, dopaminergic agents may have various effects on the urinary sphincter in PD patients at different stages. Of the 5 patients, 3 of them had early and untreated PD; the other 2 patients had long history of PD characterized by decreased response to levodopa and motor complications. Due to the limited cases at our single institute, we did not discover delayed relaxation of sphincter in PD patients with good levodopa response. At follow-up, the patients did not accept the second invasive urodynamic study. Thus, we could not evaluate the vesicosphincter function after the use of dopaminergic agents. It still requires further investigation whether this type of sphincter dysfunction is affected by the disease stage or the anti-parkinson medication.

Few studies have reported the pressure flow trace of the PD patients with the delayed relaxation of sphincter. In patients with benign prostatic obstruction, detrusor typically reaches high opening pressure, followed by a “plateau period” before the detrusor pressure drops to maintain efficient voiding. In PD patients, although detrusor underactivity is common, pressure-flow graphs usually show the “plateau period” as well, which underlies that PD patients infrequently have large PVR volume. In these 6 patients, although the detrusor pressure was high at the start of urine flow, it dropped continuously until the urine flow stopped, leading to incomplete voiding with or without large PVR volume. The continuous pressure decrease might result from sphincter bradykinesia: the sphincter was freezing before the detrusor reached the high opening pressure and the detrusor required less pressure against urethral resistance during the sluggish sphincter relaxation. Although the external sphincter bradykinesia was probably the cause, simultaneous internal sphincter (bladder neck) dysfunction could not be excluded. Similar voiding dysfunction might happen in patients with non-relaxing internal sphincter [12] or functional bladder neck contracture caused by detrusor bladder neck dyssynergia, which usually happened at the initiation of detrusor contraction and ceased at the maximum urine flow [13]. The EMG was mostly normal in patients with internal sphincter dysfunction, so it could not be discovered by EMG alone. These 6 patients could normally initiate detrusor contraction, but the detrusor could not maintain appropriate pressure and contraction time, impacting the voiding efficiency or even causing large PVR volume. Thus, this pressure flow trace pattern might also stand for a special type of detrusor underactivity. The prefrontal cortex, the insula, the hypothalamus, the periaqueductal grey and pontine micturition centre (PMC) are associated with voiding [14]. Animal models showed PMC owned a subgroup of neurons able to relax the urethral sphincter to facilitate voluntary voiding, while another subgroup of neurons could prolong the contraction [15]. If this is comparable to human, PMC degeneration could partly disclose the mechanism underlying this distinctive phenomenon.

MSA progresses fast and has a poor prognosis, and it is often easily confounded with PD. Of note, patient 1 had a large PVR volume at early stage, which might suggest MSA. He had a 6-month history of left upper limb resting tremor without rigidity and bradykinesia. During follow up, he presented masked face and the tremor progressed to left upper and lower limbs but still minimal bradykinesia. 18F-AV-133 positron emission tomography showed mild loss of dopaminergic neurons, supporting the diagnosis of PD. The anal sphincter needle EMG was done separately by a neurologist but did not show neurogenic changes. Although the patient had a large amount of PVR both at the time of the initial urodynamic study and follow-up, he denied voiding difficulty or urinary incontinence. Early voiding dysfunction alerts the diagnosis of MSA, but the patient showed slow progression on movement symptoms or LUTS during the follow-up period. Neither did he present other autonomic dysfunction, so the diagnosis of MSA cannot be made. Other 3 patients (patient 2,4 and 5) ever received external anal sphincter EMG during their disease course, but the results did not show featured changes that were frequently seen in MSA. Pavlakis et al. believed that sphincter bradykinesia was peculiar for patients with PD [8]. We did not discover this abnormality in patients with Parkinsonism-plus syndrome. Sakakibara et al. reported that open bladder neck at the beginning of filling cystometry was unique for MSA [16]. Similarly, if the delayed sphincter relaxation was unique for PD, it might be a clue of distinguish PD from other Parkinsonism-plus syndromes like MSA in some early or equivocal cases. Long term close follow-up is required in patients with early PD.

The limitation of this study is its retrospective design and limited sample size, although we reviewed the patients during the 7-year study period. And it was not uncommon that some patients were excluded due to the diagnosis revision into Parkinsonism-plus syndrome, especially MSA. Once a patient is identified having the delayed sphincter relaxation, the patients may be recruited for prospective study to learn more about its clinical significance and its specificity in PD. Given the rarity of the delayed sphincter relaxation, it needs to be questioned whether the 6 patients we studied can generally reflect the nature of this abnormality. Also, a video urodynamic study with concomitant needle sphincter EMG is the most desirable way to evaluate the neurogenic LUTS, but we did not routinely perform this complicated procedure in daily clinical practice. EMG by surface electrodes is non-invasive and easy to perform. However, it does not represent the specific sphincter, and the evidence of using this method to diagnose sphincter dysfunction is weak because of its low sensitivity [17]. Therefore, EMG by surface electrodes might miss some positive cases. Meanwhile, in PD patients with BPH, the sphincter dysfunction may be easily neglected. It should draw attention if transurethral resection of prostate is planned for these patients.

## Conclusions

The delayed urinary sphincter relaxation is a rare but repeatable phenomenon in patients with PD. It seldom causes disturbing voiding dysfunction, as reported by the patients, and does not progress prominently. Whether the delayed urinary sphincter relaxation is unique for patients with PD and its role in distinguish PD from Parkinsonism-plus syndrome needs long term study with more cases. Patients should be prospectively recruited once identified to understand the nature of this special sphincter dysfunction.

## Supplementary Information

**Additional file 1:** An example of pseudo-detrusor sphincter dyssynergia. The file shows a pressure flow trace of a 72-year-old male patient with a history of Parkinson’s disease for 10 years.

## Abbreviations

PD: Parkinson’s disease
LUTS: lower urinary tract symptoms
MSA: multiple system atrophy
BOO: bladder outlet obstruction
PVR: post void residual
BPH: benign prostatic hyperplasia
DSD: detrusor sphincter dyssynergia
BOOI: bladder outlet obstruction index
IPSS: International Prostate Symptom Score
EMG: electromyography
BCI: bladder contractility index
LEDD: levodopa equivalent daily dose
DO: detrusor overactivity
PdetQmax: detrusor pressure at maximum urine flow
Qmax: maximum urine flow;
PMC: pontine micturition centre
