# Supplementary material for: Evaluation of neurogenic bladder outlet obstruction mimicking sphincter bradykinesia in male patients with Parkinson’s disease
Source: BMC Neurol. 2021 Mar 19;21:125. doi: 10.1186/s12883-021-02153-4 (PMC7977181; doi:10.1186/s12883-021-02153-4)
Supplement: Supplementary file 1 — Additional file 1: An example of pseudo-detrusor sphincter dyssynergia. The file shows a pressure flow trace of a 72-year-old male patient with a history of Parkinson’s disease for 10 years. [file 12883_2021_2153_MOESM1_ESM.pdf]

Title: Evaluation of neurogenic bladder outlet obstruction mimicking sphincter bradykinesia in male patients with Parkinson's disease

Authors: Tianying Xing[a], Jinghong Ma[b], Tongwen Ou[a]

Affiliations:

a. Department of Urology, Xuanwu Hospital, Capital Medical University, Beijing 100053, PR China

b. Department of Neurology, Xuanwu Hospital, Capital Medical University, Beijing 100053, PR China

Correspondence to Tongwen Ou

No.45 Changchun Street, Xicheng, Beijing 100053, PR China

Telephone: +86-10-83198388

Email: tongwenou67@sohu.com

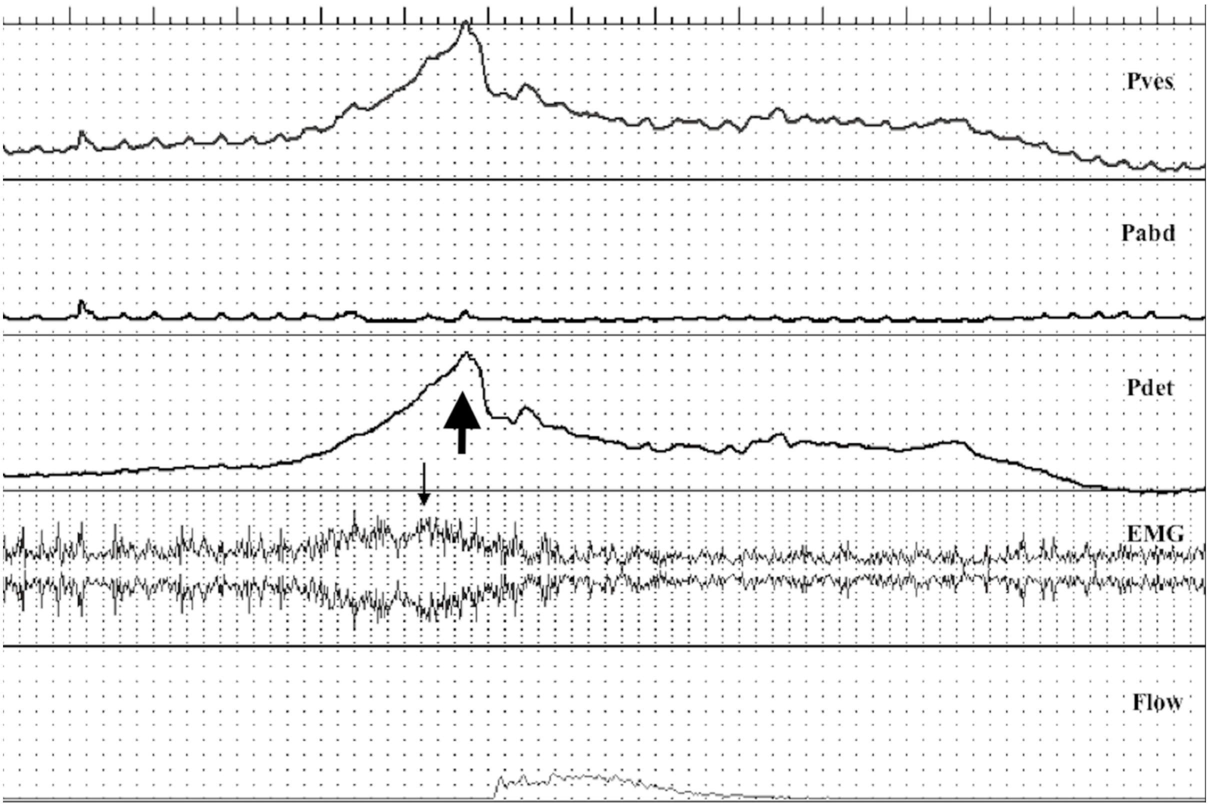

Additional Figure An example of pseudo-detrusor sphincter dyssynergia

The figure shows a pressure flow trace of a 72-year-old male patient with a history of Parkinson's disease for 10 years. The patient had terminal detrusor overactivity. The EMG sound augmented because the patient contracted pelvic floor muscles voluntarily to avoid urine leakage (arrow). But the urge was too strong and the permission to void was given (bold arrow). Sphincter relaxed properly as the EMG sound decreased. Detrusor pressure dropped after relaxation but it did not drop evenly and continuously after the peak.

Pves: Intravesicle pressure; Pabd: Abdominal pressure; Pdet: Detrusor pressure; EMG: Electromyography.
